# Learning from Women with a Body Mass Index (Bmi) ≥ 30 kg/m^2^ who have Breastfed and/or are Breastfeeding: a Qualitative Interview Study

**DOI:** 10.1007/s10995-018-2679-7

**Published:** 2019-01-04

**Authors:** Stephanie Lyons, Sinéad Currie, Debbie M. Smith

**Affiliations:** 1 Centre for Health Psychology, Division of Psychology and Mental health, School of Health Sciences, Faculty of Biology, Medicine and Health, University of Manchester, Manchester, M13 9PL UK; 2 Faculty of Natural Sciences, University of Stirling Psychology, University of Stirling, Stirling, Scotland FK9 4LA UK; 3 School of Social and Health Sciences, Leeds Trinity University, Leeds Trinity University, SB37 Jervaulx Hall, Brownberrie Lane, Horsforth, LS18 5HD UK

**Keywords:** Breastfeeding, Experiences, Obesity, Body mass index

## Abstract

*Objectives* Women with a BMI ≥ 30 kg/m^2^ are less likely to initiate and maintain breastfeeding compared to women with a BMI ≤ 30 kg/m^2^. Reasons for this disparity are not understood. Therefore, this qualitative interview study aimed to learn from women with a BMI ≥ 30 kg/m^2^ who have breastfed. *Methods* Eighteen women participated in a semi-structured telephone interview. Participants were required to have had a BMI ≥ 30 kg/m^2^ at the start of their pregnancy, and have breastfed and/or be currently breastfeeding. An inductive thematic analysis was used to analyze data.* Results* Two themes were identified: ‘personal control over breastfeeding behavior’ and ‘realistic expectations of the breastfeeding journey’. To achieve their breastfeeding goals, women described the importance of feeling in control of their behaviors, and having realistic expectations, when facing social and practical barriers. They gained this control and formed realistic expectations by seeking support and information. In particular, gaining support from other breastfeeding women with a BMI ≥ 30 kg/m^2^, and information about alternative positioning, and compatible clothing and nutrition helped women to breastfeed.* Conclusions for Practice* Having adequate information and support in order to feel in control of breastfeeding behavior and form realistic expectations are vital contributors to breastfeeding behaviors in women with a BMI ≥ 30 kg/m^2^. Future work is necessary to develop suitable interventions and to investigate their feasibility.

## Significance

*What is already known on this subject?* Women with a BMI ≥ 30 kg/m^2^ are less likely to breastfeed than women with a BMI ≤ 30 kg/m^2^. Previous research has focused on identifying the barriers which prevent these women breastfeeding, but there has been little investigation of contributors to breastfeeding behavior. *What this study adds?* Women with a BMI ≥ 30 kg/m^2^ reported that having control over their breastfeeding behavior and gaining realistic expectations helped them to overcome social and practical barriers (such as positioning for social feeding) and breastfeed. These findings inform current care and intervention development, in order to better support breastfeeding behaviors.

## Introduction

Breastfeeding has numerous benefits for woman and child, including reducing the risk of serious illness (Horta et al. 2015; Luan et al. 2013). Therefore, the World Health Organization (WHO) recommends that all women should initiate and maintain exclusive breastfeeding for the first 6 months, and continue with complementary breastfeeding until the child is at least 2 years (WHO 2016).

Despite global variation in breastfeeding rates (UNICEF 2016), consistently fewer women with a Body Mass Index (BMI) ≥ 30 kg/m^2^ than normal-weight women (i.e. BMI between 18 and 24.99 kg/m^2^) follow the WHO breastfeeding recommendations. For example, women with a BMI ≥ 30 kg/m^2^ are less likely to initiate (e.g. 82.2% vs. 86.4%), and to breastfeed for at least 6 months (e.g. 44.4% vs. 53.8%; Jarlenski et al. 2014). With the number of women with a BMI ≥ 30 kg/m^2^ who are of childbearing age (i.e. 16–44 years) steadily rising (Public Health England 2015), and the amplified importance of breastfeeding for this population (e.g. increased postpartum weight loss, reduced risk of childhood obesity; Vinter et al. 2014; Yan et al. 2014), develop an understanding of how this population makes infant feeding decisions, and why initiation and maintenance is low, is vital in order to better support breastfeeding behaviors.

The majority of the previous research conducted in this area has focused on the identification of barriers that prevent initiation and maintenance. For example, studies have shown that women with a BMI ≥ 30 kg/m^2^ lack confidence in their ability to reach breastfeeding goals, experience challenges with latching and positioning, find it difficult to find suitable clothing for breastfeeding, and have few friends or family members who have breastfed (Garner et al. 2016; Hauff et al. 2014). Furthermore, a recent review highlighted several mechanical (e.g. larger breasts), biological (e.g. delayed lactogenesis II) and psychosocial factors (e.g. low confidence, self-efficacy, body image) that may impede breastfeeding behaviors (Babendure et al. 2015). However, we currently know very little about what can contribute to and support breastfeeding initiation and maintenance in women with a BMI ≥ 30 kg/m^2^.

One way of identifying and understanding these factors is to explore the experiences of women with a BMI ≥ 30 kg/m^2^ who have breastfed. Such exploration requires a qualitative approach, in order to generate person-centered descriptions of their breastfeeding journeys (Padgett 2016). The current qualitative study aims to explore and learn from women with a BMI ≥ 30 kg/m^2^ who had breastfed and/or were currently breastfeeding by exploring their views and experiences. The findings aid the literature base in terms of what supports these women to breastfeed and inform future research and care, to ultimately better support breastfeeding initiation and maintenance for women with a BMI ≥ 30 kg/m^2^.

## Methods

### Study Design

To guide the research focus and aims, two patient and public involvement sessions (PPI) were conducted with women who had a BMI ≥ 30 kg/m^2^ and had breastfed. Seven women were recruited through an online advertisement (i.e. posted on several breastfeeding-related social media pages and mailing lists). Some members of the PPI group helped to design the study and the study materials including the interview topic guide. Ethical approval was gained from the University Research Ethics Committee (Ref: 15,453). COREQ criteria (Tong et al. 2007) was followed to generate this manuscript.

### Interviews

Interviews were conducted with women who were situated in the North-West of England between December 2015 and March 2016. North-West England is a deprived area, with high obesity and low breastfeeding rates (HSCIC 2016; NHS 2017). Women were eligible if they reported having had a BMI ≥ 30 kg/m^2^ at the start of their pregnancy, and if they had breastfed in the past, or were currently breastfeeding at the time of the interview. Due to the exploratory nature of this study, these criteria were the only necessary for participation. Participants were recruited through two routes; (1) a standardized online advertisement was posted on several North-West England breastfeeding forums and social media breastfeeding groups, and (2) an individual email was sent to women who had previously volunteered for the patient and public involvement (PPI) group and had expressed interest in participating in future research. If interested, women were asked to email the researchers to set an interview.

Interviews were conducted via the telephone, a method preferable to women with young children due to its flexibility (Trier-Bieniek 2012). Likewise, this method was appropriate for the analysis, as it encourages a participant-centered approach, and allows participants to remain relatively anonymous, which can facilitate the generation of rich data (Trier-Bieniek 2012). A semi-structured topic guide developed from past literature and the PPI sessions was used, in order to explore and learn from the women’s experiences (Table 1). A pilot interview was conducted to ensure the acceptability of the topic guide prior to use with the sample, but no changes were made.

**Table 1:** Interview topic guide

Topics	Questions
Experience of initiating breastfeeding	Did you intend to breastfeed? Why? When did you decide?
	What are the reasons why you initiated breastfeeding?
	How was the experience of initiation of breastfeeding after birth?
	Did you feel prepared to initiate breastfeeding?
Views about BMI ≥ 30 kg/m^2^ and initiating breastfeeding	What do you think helps women with a BMI of 30 or more initiate breastfeeding?
	What do you think stops women with a BMI of 30 or more initiating breastfeeding?
Maintaining breastfeeding	What were the reasons why you maintained breastfeeding?
Views about BMI ≥ 30 kg/m^2^ and maintaining breastfeeding	What do you think helps women with a BMI of 30 or more maintain breastfeeding?
	What do you think stops women with a BMI of 30 or more maintaining breastfeeding?
Support and invitation for additional comments	What help do you think women with a BMI of 30 or more need to initiate and maintain breastfeeding?
	Is there anything else that you want to add?

After contacting the researcher, eligible women (N = 29) were sent a participant information sheet and consent form to gain informed consent. Twenty-one women returned their consent forms, and twenty interviews were scheduled. At the time of their interviews, two women were unable to take part or rearrange. All interviews were conducted by the same researcher (SL). This researcher was an MSc student, and was given training and support on interview technique by DS. Interviews were audio-recorded and anonymized whilst being transcribed verbatim.

### Data Analysis

Data were analyzed using inductive thematic analysis, according to the steps stated by Braun and Clarke (2006; Table 2). Two researchers (SL and DS) completed the coding process independently (i.e. steps 1–3), and then discussed and agreed themes (i.e. steps 4–5). As little research had focused on contributors to breastfeeding behaviors, thematic analysis was an appropriate method for the research question, allowing flexibility and freedom from theoretical constraints (Braun and Clarke 2006). Analysis was conducted alongside data collection, and after the fifteenth interview, the researchers felt that no new information was emerging. A further three interviews were conducted to ensure no new data emerged, and that the sample size was appropriate to summarize the experience of the women. Once the report had been written, two PPI members reviewed it to ensure they felt it reflected their experience, increasing the rigor of the results (Padgett 2016). Their feedback was used to enhance the clarity of the theme descriptions in the report (step 6).

**Table 2:** Descriptions of the steps of analysis

Step	Description
1	Researchers immersed themselves in the data set, by reading and re-reading the interviews
2	Interesting words and phrases were highlighted and assigned meanings on each transcript, and were then grouped semantically, leading to the generation of initial codes. The coding process was carried out with the view of identifying contributors to breastfeeding behaviours
3	Semantically similar codes were combined developing potential themes (i.e. underlying patterns running throughout the data)
4	Potential themes were reviewed, ensuring that the themes represented the data set
5	The themes were refined and given clear definitions and names
6	The report was sent to two PPI members

## Results

**Fig. 1:**
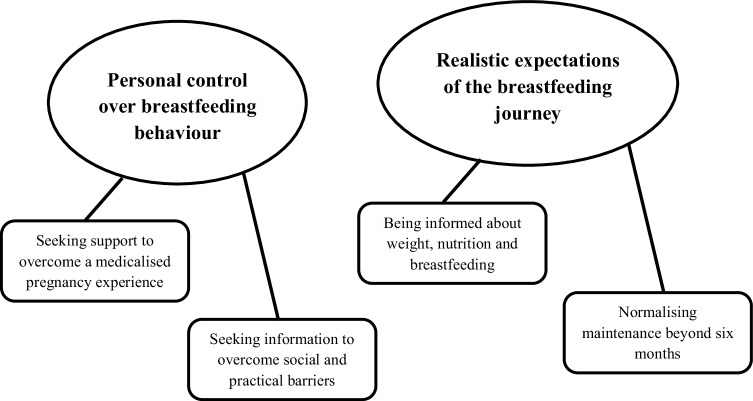
Themes and subthemes

Eighteen women participated in telephone interviews (mean interview length = 41.56 min, *SD* = 8.32 min). All had intended to breastfeed prior to their child’s birth. Women ranged in self-reported breastfeeding experience from 4 months to 3 years. Two themes are presented; ‘personal control over breastfeeding behavior’, and ‘realistic expectations of the breastfeeding journey’ (Fig. 1). The themes represent the contributors to breastfeeding behaviors, and the subthemes explain how they achieved them. Excerpts are presented for each theme to facilitate judgements of trustworthiness (Table 3). As first-names can often be associated with particular attributes (Gebauer et al. 2012), pseudonyms were generated for each participant using a random name generator.

**Table 3:** Excerpts to support each subtheme

Theme	Subtheme	Quotes
1. Personal control over breastfeeding behaviour	1a. Seeking support to overcome a medicalised pregnancy experience	“Throughout the process of being pregnant there is, if you have high BMI, there’s a constant sort of you are high risk, your body is less than ideal for giving birth, and so it sort of like sends a message like you can’t do this or it’s going to be harder for you, you know there’s something wrong with you, so I think for some people that could get into their heads in terms of carrying on after the birth and thinking I shouldn’t” (Catherine) “She was like you need to go to the local breastfeeding support group you know you’ll get support from women like you and it’ll, it’ll help your confidence, and it was the best thing I did, definitely” (Chloe)
	1b. Seeking information to overcome social and practical barriers	“A lot of men will have the impression that erm if you’re a young fit girl then that’s okay to get your boobs out you know they’ll have a look at that that’s fine, but I think erm it’s unacceptable when you’re not a skinny person, you know it’s not seen as acceptable to be getting your boobs out”(Clare) “They [breastfeeding peers] were really really good for those types of tips like how you could, you know, do different positions that might help so they were a lot more practical from that kind of view, and I don’t think you get that kind of, that kind of support when you know you’re in the hospital”(Bethany)
2. Realistic expectations of the breastfeeding journey	2a. Being informed about weight, nutrition and breastfeeding	“I was also told it’s very important to eat whilst you’re breastfeeding especially within the first few weeks so every time you breastfeed your child have a biscuit” (Ebony) “I did look into it and looked exactly into what how many other calories I could or should have and where they should come from and the food I should and shouldn’t be eating so, yea I sort of, I sort of adjusted my expectations”(Eve)
	2b. Normalising maintenance beyond 6 months	“All the literature and everything was all geared up for like 6 months isn’t it, obviously you hear like the World Health Organisation says 2 years but pretty much everything, society, pretty much everything is geared up to 6 months and then you’ve kind of done you’re bit, past 6 months you’re kind of getting the ‘how long are you gonna be feeding him for?’, after a year they’ve just realised you’re slightly weird” (Bethany) “I help run the sling library so within that environment there’s a lot of people who practice extended breastfeeding or full term or whatever of the many other ways of describing it, so erm I I don’t really feel like unusual for doing it” (Ruby)

### Theme 1—‘Personal Control Over Breastfeeding Behavior’

Women viewed breastfeeding as a natural behavior. However, after being deemed ‘high risk’ by health professionals during pregnancy (i.e. regardless of their whole health status), and requiring medical intervention during pregnancy or labor, they did not feel in control of their feeding decisions, which negatively impacted upon breastfeeding initiation and maintenance. Furthermore, social and practical barriers due to their body size and shape reinforced the idea that breastfeeding was not for them. However, additional support and tailored advice helped the women normalize breastfeeding and regain control of their feeding choices, therefore, supporting initiation and maintenance. Two subthemes are presented: ‘seeking support to overcome a medicalized experience’ and ‘seeking information to overcome social and practical barriers’.

### Seeking Support to Overcome a Medicalized Pregnancy Experience

This subtheme represents how being labelled as ‘high risk’, and experiencing BMI-related complications (e.g. gestational diabetes, pre-eclampsia) can lead women to feel as though they have little control during their pregnancy, which can negatively impact upon breastfeeding behaviors. For example, many of the women described how their risk status, and how their status was communicated to them, had removed their sense of control during pregnancy. As breastfeeding was often described as a ‘natural’ behavior, this medicalized experience also led them to expect breastfeeding to be more difficult. Furthermore, as many of the women had indeed experienced complications during their pregnancy and birth (i.e. in particular complications which delayed breastfeeding initiation, such as emergency cesarean sections and babies being admitted to neonatal intensive care), their perceived lack of control over their breastfeeding was often reinforced.

The women described how they attempted to regain control to overcome these challenges by actively seeking support. For example, many women acknowledged that they had needed a ‘cheerleader’, and that seeking professional and peer support had provided encouragement and reassurance that breastfeeding was for them. Therefore, promoting control over feeding practices despite pregnancy risk status, and helping women to find appropriate support may increase breastfeeding in this population.

### Seeking Information to Overcome Social and Practical Barriers

Social and practical barriers to breastfeeding were discussed, and women highlighted how they can lead to the perception that breastfeeding is not a suitable infant feeding choice for women with a BMI ≥ 30 kg/m^2^, reinforcing the perceived lack of control. Many of the women described how they felt that society expected women with a BMI ≥ 30 kg/m^2^ to remain covered when breastfeeding, which makes public breastfeeding challenging. This belief was reinforced by the absence of pictures of women with higher BMIs in the breastfeeding literature, and the lack of nursing/breastfeeding clothes available in larger sizes. Furthermore, practical barriers, such as larger breast and body size, led to difficulty when attempting to breastfeed in the positions frequently demonstrated to them by health professionals (e.g. cradle, cross-over), and concern that they may suffocate their child whilst breastfeeding.

Women described how seeking information about where to find nursing clothes in appropriate sizes, and better positioning for women of different sizes and shapes, helped them to regain control. However, the women also expressed that, during antenatal care, this type of information was often ignored in preference of delivering a ‘sales pitch’ on the health benefits associated with breastfeeding, and that they had actively sought this information through internet searches and asking their peers at breastfeeding support groups.

### Theme 2—‘Realistic Expectations of the Breastfeeding Journey’

Having realistic expectations of the breastfeeding journey helped the women initiate and maintain breastfeeding. Women felt that because having a BMI ≥ 30 kg/m^2^ can lead to increased concerns about nutrition (i.e. adequacy of their milk) and losing postnatal weight, they had more unrealistic expectations about the implications of breastfeeding. Furthermore, health professional’s focus on latching as a measure of breastfeeding success can make it difficult for women with a BMI ≥ 30 kg/m^2^ to access the information and support they need to maintain breastfeeding beyond 6 months. However, having realistic expectations was reflected by the women as reducing confusion, worry and disappointment, and ultimately increasing breastfeeding. Two subthemes were identified: ‘being informed about weight, nutrition and breastfeeding’ and ‘normalizing maintenance beyond 6 months’.

### Being Informed About Weight, Nutrition and Breastfeeding

Overcoming concerns, and gaining realistic expectations of weight and nutrition whilst breastfeeding can help women with a BMI ≥ 30 kg/m^2^ to initiate and maintain. For example, some women discussed the belief that diet content is important for their milk quality, in that the nutrition a child receives is wholly determined by the type of food the mother consumes, and, therefore, that women who held these beliefs, but did not perceive their diets as adequately nutritious, would not initiate breastfeeding. Some women also acknowledged the role of society and health professionals in promoting these unhelpful beliefs, perpetuating the problem. This issue was further complicated by the perception that breastfeeding mums need to eat more. For example, many women expressed how they were encouraged to increase their calorie intake in order to maintain their milk supply. These conflicting messages can lead women to feel confused and worried about the impact of their diet on their milk, and the types and amounts of foods they should be consuming to maintain an adequate supply. Similarly, the majority of women also expressed that the expectation of weight loss was an important incentive to initiate and maintain breastfeeding. However, many of the women felt that breastfeeding had actually delayed their weight loss, either because they were eating more, or because they believed reducing their calorie intake would reduce their milk supply, which could have had a negative impact on breastfeeding.

The women, therefore, described how being informed had helped them to overcome their concerns. For example, actively searching the internet, and asking health professionals and peers had helped women to form realistic expectations about the impact of their diet on their milk, and of the impact breastfeeding would have on weight loss. Women highlighted forming realistic expectations as a vital process for avoiding cessation when their previously unrealistic expectations were not met. As this information was not distributed as a part of routine care, doing so may be particularly useful for women with a BMI ≥ 30 kg/m^2^.

### Normalizing Maintenance Beyond 6 Months

For women with a BMI ≥ 30 kg/m^2^, successful breastfeeding is a journey that lasts beyond just a ‘good latch’. However, women felt that health professionals focus their attention on the physical aspects of breastfeeding behavior, and believed support to be minimal once latching is accomplished; women often felt that health professionals’ expectations of breastfeeding duration did not match their own. For example, many of the women felt that the WHO guideline of exclusive breastfeeding for 6 months is often perceived as an end point, and expressed that they felt under pressure from others, including their family, friends, and health professionals to stop breastfeeding once their child had reached this point. The women stressed how shifting this focus from latch to maintenance in order to normalize breastfeeding beyond 6 months, and provide realistic expectations of the length of their breastfeeding journey, was vital for success. The majority of women commented on how being exposed to, and receiving support from other mums who were maintaining breastfeeding had helped them to access this information, gain realistic expectations and normalize maintenance. Therefore, ensuring women know where to access this support may help them to maintain breastfeeding and normalize their views of breastfeeding.

## Discussion

The aim of this study was to explore and learn from the views and experiences of women with a BMI ≥ 30 kg/m^2^ who had breastfed and/or were currently breastfeeding. The two themes identified provide insight into contributors of breastfeeding initiation and maintenance in this population, and, therefore, can inform future interventions and ante- and postnatal care.

Consistent with the general population, women with a BMI ≥ 30 kg/m^2^ view breastfeeding as a natural part of motherhood (Schmied and Barclay 1999). However, for this population, the high level of medical intervention due to their BMI (Kerrigan et al. 2015), and the numerous social and practical barriers, could lead to a perceived lack of control over their ability to breastfeed. These findings are supported by recent qualitative studies that investigated the barriers to breastfeeding in this population, finding that medical intervention and difficulty finding suitable breastfeeding clothing had a negative impact on breastfeeding (Garner et al. 2016; Keely et al. 2015). As perceiving a behavior is within one’s control is a crucial factor of whether a behavior is subsequently performed (Bandura 1986), perceiving a lack of control over infant feeding behaviors may explain why many women with a BMI ≥ 30 kg/m^2^ do not initiate and maintain breastfeeding.

However, women described how seeking additional support and information had helped them to normalize breastfeeding and regain control over their feeding choices, ultimately helping them to engage in breastfeeding behaviors. Therefore, providing women with this support and advice, rather than relying on them actively seeking it, may have a positive impact on breastfeeding behaviors. For example, as studies have shown that women with a BMI ≥ 30 kg/m^2^ often intend to breastfeed for similar durations as women with a BMI ≤ 30 kg/m^2^ (Hauff et al. 2014), this provision may help them to translate their intentions into action by overcoming the barriers associated with seeking support (e.g. believing the groups are only for ‘successful breastfeeders’; Keely et al. 2015). As the women in this study highlighted that breastfeeding support groups were a particularly useful source of this information and support, we recommend that the benefits of attending a local group is discussed with all women with a BMI ≥ 30 kg/m^2^. All recommendations can be found in Table 4.

**Table 4:** Recommendations made for health professionals and practice

Recommendations
Health professionals should discuss the benefits of attending a local breastfeeding support group with all women with a BMI ≥ 30 kg/m^2^
Health professionals should undergo additional training, to increase their awareness of the needs of women with a BMI ≥ 30 kg/m^2^, and expand their knowledge of positioning and nutrition, specific to this population

Again, consistent with the general population, having realistic expectations of the breastfeeding journey for these women is vital for initiation and maintenance (Gregory et al. 2015). However, a conflict between health professionals’ focus during care and the women’s needs was found. Psychological theory posits outcome expectancies as important determinants of behavior (Bandura 1986), and therefore may explain why few women with a BMI ≥ 30 kg/m^2^ initiate and maintain breastfeeding. However, as these women overcame this conflict (e.g. by searching independently for further information and support) and did breastfeed, two recommendations are made. The first is that, again, that the benefits of attending breastfeeding support groups are discussed, as the information and support they require is often available here. The second is that health professionals undergo additional training, to increase their awareness of the needs of women with a BMI ≥ 30 kg/m^2^, and expand their knowledge of positioning and nutrition, specific to this population. This training would attempt to ensure that women who were uncomfortable, disinterested or unable to attend breastfeeding support groups could access this information and support from their health professional. As health professionals often find caring for women with a BMI ≥ 30 kg/m^2^ challenging (Garner et al. 2014), and lack this population-specific information (Rasmussen et al. 2006), it would also attempt to ensure they felt comfortable and confident in their ability to provide the necessary information and support.

Future intervention research is necessary to investigate the possibility of utilizing the identified factors in order to increase breastfeeding success in this population. Psychological theory can explain and predict behavior change, and, therefore, Social Cognitive Theory (Bandura 1986) and the Theory of Planned Behavior (Ajzen and Fishbein 1980) offer useful suggestions as to how we can use this knowledge to support breastfeeding behaviors. For example, increasing role mastery (i.e. learning to perform the appropriate breastfeeding positions), vicarious experience (i.e. learning by seeing others successfully breastfeeding) and promoting realistic outcome expectancies, should increase the women’s sense of control and confidence (Bandura 1977), and ultimately, breastfeeding initiation and maintenance. Self-efficacy has been shown to increase breastfeeding behaviors in the general population (Nichols et al. 2007). Also, normalizing breastfeeding for women with a BMI ≥ 30 kg/m^2^ (e.g. by providing normative feedback) should also increase initiation and maintenance (Ajzen and Fishbein 1980; Lawton et al. 2012). However, as healthcare professionals would likely need to deliver such interventions, future research that incorporates both psychological and clinical expertise is vital.

This study has some limitations. All the women were residing in one region of England (North-West), meaning that the results only reflect the breastfeeding initiatives and support within this region. However, this is a deprived area, with high obesity and low breastfeeding rates (Baker 2018; Department for Communities and Local Government 2015; Department of Health 2013). Therefore, we believe that women in this region would be representative of other women with a BMI ≥ 30 kg/m^2^. The sample was limited to those participants who volunteered, increasing the risk of selection bias (Robinson 2014). Furthermore, although conducting interviews via telephone has been criticized for shortening interview length (Irvine et al. 2012), the mean interview length in this study (i.e. 42 min) suggests that this method did not negatively impact the results. We should also acknowledge that media coverage about breastfeeding, particularly breastfeeding beyond 6 months, is often negative, presented in a sexualized context which makes others uncomfortable (Brodribb 2015; Foss 2013), which could partially explain the importance which the women placed on control, confidence, information and normalizing.

However, this study also had several strengths, including the PPI involvement in the study design, and conducting a member check of the full report, improving its quality (Callard et al. 2012; Padgett 2016). Furthermore, to our knowledge, this study is the first of its kind to investigate what can be learned from women with a BMI ≥ 30 kg/m^2^ who have breastfed, rather than focusing on identifying barriers, offering a unique opportunity to investigate contributors to breastfeeding behaviors in this population.

In conclusion, this study aimed to learn from the views and experiences of women with a BMI ≥ 30 kg/m^2^ who had breastfed and/or were currently breastfeeding, and found that feelings of control and having realistic expectations were important contributors of behavior. As breastfeeding has amplified importance in this population, we recommend that future research should utilize the proposed psychological frameworks to develop acceptable and feasible interventions, and health professionals should undergo further training, and discuss the benefits of attending breastfeeding support groups, in order to better support breastfeeding behaviors.
